# An epitope-specific chemically defined nanoparticle vaccine for respiratory syncytial virus

**DOI:** 10.1038/s41541-021-00347-y

**Published:** 2021-06-18

**Authors:** Armando Zuniga, Oliver Rassek, Melissa Vrohlings, Aniebrys Marrero-Nodarse, Kerstin Moehle, John A. Robinson, Arin Ghasparian

**Affiliations:** 1 Virometix AG, Schlieren, Switzerland; 2 Chemistry Department, University of Zurich, Zurich, Switzerland; 3 Present Address: Shape Biopharmaceuticals Inc, Cambridge, MA USA; 4 Present Address: CDR-Life, Schlieren, Switzerland

**Keywords:** Peptide vaccines, Viral infection

## Abstract

Respiratory syncytial virus (RSV) can cause severe respiratory disease in humans, particularly in infants and the elderly. However, attempts to develop a safe and effective vaccine have so far been unsuccessful. Atomic-level structures of epitopes targeted by RSV-neutralizing antibodies are now known, including that bound by Motavizumab and its clinically used progenitor Palivizumab. We developed a chemically defined approach to RSV vaccine design, that allows control of both immunogenicity and safety features of the vaccine. Structure-guided antigen design and a synthetic nanoparticle delivery platform led to a vaccine candidate that elicits high titers of palivizumab-like, epitope-specific neutralizing antibodies. The vaccine protects preclinical animal models from RSV infection and lung pathology typical of vaccine-derived disease enhancement. The results suggest that the development of a safe and effective synthetic epitope-specific RSV vaccine may be feasible by combining this conformationally stabilized peptide and synthetic nanoparticle delivery system.

## Introduction

Respiratory syncytial virus (RSV) can cause acute lower respiratory tract infections that often require hospitalization, in particular during infancy, childhood, and in the elderly. Natural infections with RSV typically elicit a short-lived immune response that does not protect from future re-infections^1^. RSV contains a single-stranded negative-sense RNA and has a lipid bilayer envelope^2^. The viral G and F membrane glycoproteins have important roles in cellular entry by mediating the fusion of the viral and host cell membranes. The trimeric F-glycoprotein mediates fusion by first binding to cellular entry receptors, including insulin-like growth factor I and human nucleolin^3^. However, the complete biochemical mechanism of RSV cellular entry at the atomic level is so far incompletely understood^4^.

A licensed RSV vaccine is not yet available and the search for a vaccine has been plagued by problems with the stability, production, and potency of candidates and a long history of vaccine-associated enhanced respiratory disease (VAERD). VAERD was first observed in the mid-1960s during a phase III trial of a formalin-inactivated RSV (FI-RSV) vaccine, in which infants immunized with FI-RSV experienced enhanced respiratory disease after infection with wild-type RSV^5^. The enhancement appears to be driven by induction of imbalanced cell-mediated immune responses and non-neutralizing antibodies (nAbs) to coat proteins that enhance viral uptake into host cells via Fcγ receptors^6–8^. Recent structural insights into the trimeric F-glycoprotein reveal a complex conformational landscape that includes a prefusion conformation required for viral entry and a rearranged trimeric post-fusion conformation. Without stabilization, the pre-F ectodomain flips spontaneously to the post-F form, which elicits more non-nAbs than the pre-F form^9^.

A prophylactic humanized murine monoclonal nAb called palivizumab (Synagis) became available for high-risk infants in the early 2000s^2,10^. Palivizumab binds an epitope within antigenic site II (FsII) that is preserved in both the pre- and post-fusion F conformations. However, the cost and relatively short half-life of this monoclonal antibody (mAb) have limited its wider use and it is not approved for adults or general pediatric use.

Progress in structural studies of the F-glycoprotein and its complexes with nAb fragments have recently enabled structure-based approaches to RSV vaccine design. The crystal structure of an affinity-matured variant of palivizumab called Motavizumab^11^ revealed the epitope in a helix–turn–helix motif (PDB 3IXT)^12^. A proof of principle for an epitope-focused vaccine design was reported in 2014^13^, when the Motavizumab epitope was grafted onto a small scaffold protein, and then optimized to provide a stabilized epitope mimetic. Fusion of this mimetic to hepatitis B core antigen virus-like particles afforded a construct that failed to elicit nAbs in mice but was successful in inducing nAbs in macaques. Further successes with the structural vaccinology approach have been reported more recently, using protein engineering methods to design conformationally stable epitope mimetics as vaccine candidates^14–17^.

We explore here a complementary approach to epitope-focused vaccine design. This is based on using conformationally constrained synthetic peptides as B-cell epitope mimetics. The mimetics are conjugated to synthetic nanoparticles made from specially designed self-assembling lipopeptides that contain a T-helper epitope and a toll-like receptor (TLR) ligand. In previous work, we showed that such nanoparticles displaying conjugated epitope mimetics elicit strong epitope-specific humoral immune responses in animal models without the need for an external adjuvant^18–21^. With the crystal structure of Motavizumab bound to its epitope to guide mimetic design, we apply this approach here to the development of an RSV vaccine candidate. We describe a conformationally constrained and optimized epitope mimetic conjugated to self-assembling synthetic lipopeptides, called V-306. This vaccine construct elicits strong long-lasting RSV-neutralizing antibody responses in mice and rabbits that protect mice from RSV infection and disease enhancement in a validated preclinical RSV challenge model.

## Results

### Design and selection of a motavizumab epitope mimetic

**Fig. 1:**
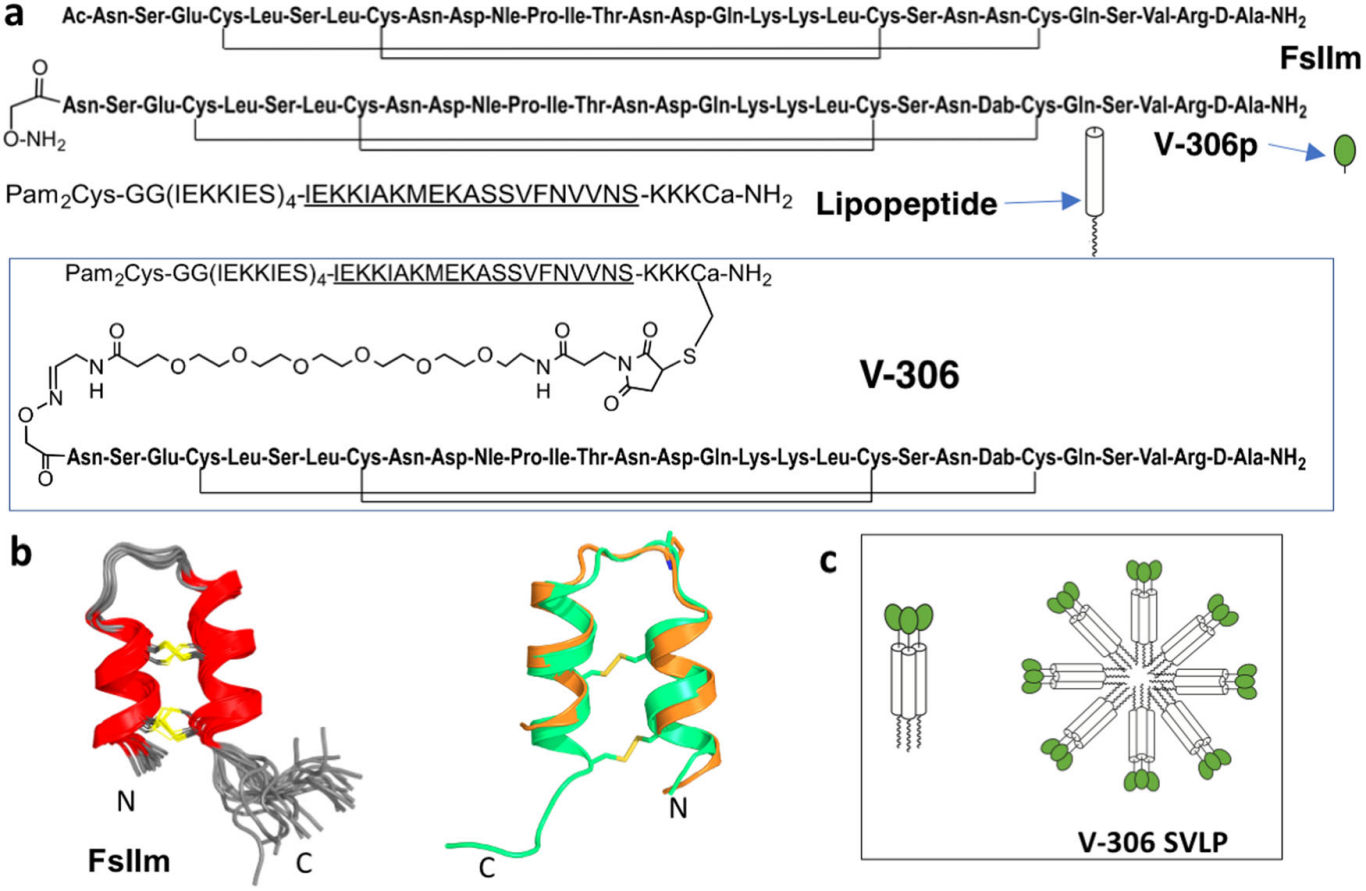
Peptide structures. **a**, i The sequences of peptides FsIIm, V-306p (Nle = l-norleucine, Dab = l-diaminobutyric acid, d-Ala = d-alanine), ii the synthetic lipopeptide (shown in single amino acid letter code, with the coiled-coil heptad repeat (IEKKIES)_4_ that forms a trimeric helical bundle, with T-helper epitope underlined, and C-terminal a = d-alanine) and, iii V-306. Pam_2_Cys is S-[*R*-2,3-bis(palmitoyloxy)propyl]-*R*-cysteine. **b**, left: Solution NMR structures of FsIIm. A superimposition of the final 20 structures is shown. Right: Superimposition of one typical NMR structure of FsIIm (green) and the Motavizumab antigen (orange) from PDB file 3IXT. **c** Schematic representation of the V-306 SVLP, formed by aggregation of trimeric coiled-coil V-306 lipopeptides (shown on the left, green ball = palivizumab epitope mimetic (V-306p), cylinder = helical coiled-coil domain, wavy line = Pam_2_Cys lipid) into a ca. 25–30-nm diameter micelle-like nanoparticle (shown on the right).

For the design of a conformationally constrained peptide mimicking the epitope recognized by Motavizumab, we used the crystal structure of the Fab fragment in complex with a peptide encompassing F-glycoprotein residues Asn254–Asn277 (PDB 3IXT)^12^. Thirteen residues in the epitope form contacts to the Motavizumab Fab, including Ser255, Leu258, Ser259, Ile261, Asn262, Asp263, Asn268, Asp269, Lys271, Lys272, Leu273, Ser275, Asn276. Sequences of F glycoproteins from palivizumab-selected mAb-resistant mutant (MARM) viruses further suggested that these positions are important for neutralization by palivizumab^22,23^. This led initially to the FsII site mimetic called FsIIm, with stabilizing sequence modifications and cysteines for cyclization via disulfide bridges at antigenically non-critical positions, shown in Fig. 1a. The solution structure of this peptide was determined by homonuclear ^1^H NMR spectroscopy (Supplementary Fig. 1). FsIIm adopts a stable helix-rich folded conformation in water (Fig. 1b). The solution structure superimposed very closely on that of the Motavizumab-bound peptide (PDB 3IXT), showing that it is an excellent structural mimetic of the epitope. Further optimization of the physical and immunological properties led to the mimetic V-306p (Fig. 1), for incorporation into a potential RSV vaccine candidate.

### Construction and structural characterization of V-306

The mimetic V-306p contains an N-terminal aminooxyacetyl group for conjugation to an engineered synthetic lipopeptide (Fig. 1a and Supplementary Fig. 2). The lipopeptide contains a promiscuous CD4^+^ T-helper epitope, a coiled-coil motif (heptad repeat IEKKIES) that forms a very stable helical trimer, and at the N-terminus the TLR-2/6 ligand Pam_2_Cys. The peptide V-306p was linked to this lipopeptide via a maleimide-PEG-aldehyde linker, to give the vaccine construct V-306 (Fig. 1a and Supplementary Figs. 2 and 3). The conjugate V-306 in phosphate-buffered saline (PBS) was analyzed by dynamic light scattering (DLS) and transmission electron microscopy (Supplementary Fig. 4). DLS revealed nanoparticle formation in PBS with a mean hydrodynamic radius (Rh) of ca. 13 nm and a polydispersity index of 0.05, consistent with the formation of highly monodisperse nanoparticles of about 26-nm diameter. Based on computer modeling and prior work^18–20^, about 60–90 copies of each V-306 lipopeptide chain should comprise each nanoparticle, with the lipid chains buried in the core of the micelle-like particle and the epitope mimetic exposed in its surface (depicted in Fig. 1c). Transmission electron microscopy also revealed the formation of nanoparticles in a similar size range 25–30 nm (see Supplementary Fig. 4).

### Peptide V-306p and V-306 bind human anti-RSV antibodies

We tested binding of V-306p and V-306 by ELISA to palivizumab and to Abs in a panel of 20 sera from convalescent RSV infected humans, with lipopeptide carrier as control (Supplementary Fig. 5). The ELISA showed specific binding of palivizumab to V-306 and V-306p, but not the lipopeptide carrier, with a lower limit of detection of 0.04 µg/ml for V-306. All 20 human sera contained Abs cross-reactive to V-306p by ELISA. We determined by ELISA titers of palivizumab-like V-306p cross-reactive antibodies ranging between 1.25 and 412 µg/ml in the human sera. The geometric mean titer of V-306p-reactive antibodies across all 20 sera was 8.14 µg/ml.

### V-306 elicits high titers of epitope-specific antibodies in mice

**Fig. 2:**
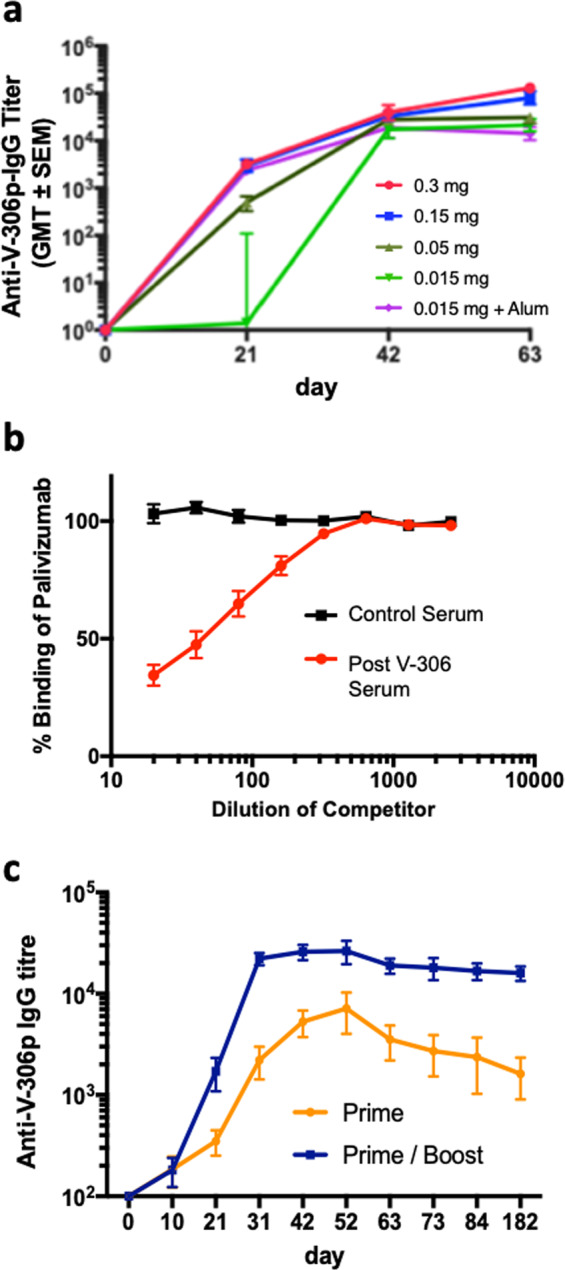
Antibody response after immunization with V-306. **a** Development of epitope-specific IgG antibodies in BALB/c mice (*n* = 6) after IM immunization on days 0, 21, and 42 with V-306 in PBS or with Adju-Phos (Alum) at the indicated doses. Shown are half-maximal binding geometric mean titers ± one SEM (GMT ± SEM) determined by ELISA using V-306p as coating antigen. **b** Competition for binding to V-306p between palivizumab and increasing concentrations of serum Abs from V-306 immunized or naive mice. **c** Longevity of Ab titers elicited in mice (*n* = 5) after one (day 0, prime) or two (days 0 and 21, prime/boost) immunizations with V-306 determined by ELISA.

We tested the immunogenicity of V-306 in BALB/c mice. Four groups of BALB/c mice (*n* = 6 animals per group) were immunized intramuscularly (IM) three times with 15, 50, 150, or 300 µg V-306 in PBS. The low dose study was also performed using Alum adjuvant (Adju-Phos^®^). Sera were analyzed by ELISA using immobilized V-306p. As shown in Fig. 2a, strong IgG responses were observed against V-306p in all dose groups already after the second immunization. Only a relatively small increase in IgG titers, relative to the second immunizations, was seen after the third immunization. The addition of Alum to the 15 µg dose improved IgG titers to the level seen with higher doses without adjuvant. We also tested by competition ELISA whether Abs elicited by V-306 in mice compete with palivizumab for binding to V-306p. The results showed significant competition between serum Abs and palivizumab binding to the antigen (Fig. 2b). To quantify the longevity of the IgG response, after vaccine prime and boost immunizations, two additional groups of BALB/c mice (*n* = 5) were immunized with V-306 in PBS one (prime) or two times (prime and boost). The kinetics of the antibody responses was monitored over a 180-day period by ELISA. The results show a detectable IgG response in both groups extending to over 6 months (Fig. 2c).

### V-306 elicits high titers of RSV-neutralizing antibodies

**Fig. 3:**
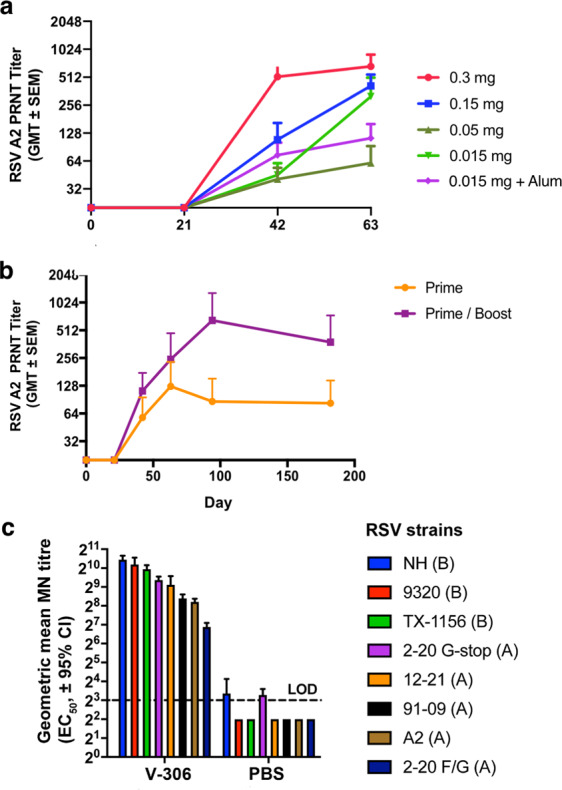
Neutralizing antibody response after immunization with V-306. **a** Development of RSV nAbs in BALB/c mice (*n* = 6) after immunization with the indicated doses of V-306 in PBS or Adju-Phos (Alum). NAbs were quantified by 60% PRNT using the RSV-A2 strain. Shown are GMT ± one SEM. Injection days 0, 21, and 42. **b** Longevity of the nAb response in mice (*n* = 5) after prime (day 0) or prime/boost (days 0 and 21) immunization. **c** Neutralization of RSV A and B strains shown by Abs in sera from rabbits (*n* = 1) immunized with either V-306 or PBS (control). The data represent the EC_50_ calculated from nonlinear regression analysis + upper limit of 95% CI. LOD limit of detection.

To quantify titers of RSV nAbs in the sera from immunized mice, a validated 60% plaque reduction neutralization test (PRNT) was performed using the RSV-A2 strain^24,25^, with geometric mean titers shown in Fig. 3a. All dose groups had detectable nAbs 3 weeks after the second immunization, which increased to high nAb titers 3 weeks after the third immunization, exceeding 10 log_2_ in some sera. The addition of Alum to the 15 µg of dose did not improve the production of nAbs after the third immunization. The longevity of the nAb response in mice sera, after a vaccine prime and boost, was also quantified by PRNT. A single injection elicited a strong antibody response (Fig. 3b). nAbs became detectable in day 42 sera. In mice immunized only once, nAb titers peaked in day 56 sera. Mice immunized two times developed high nAb titers ranging between 8 log_2_ and 11 log_2_ around day 84. Neutralization activity was detectable in both groups until the end of the observation period. These results highlight the potential of V-306 to elicit strong, long-lasting nAb responses.

The immunogenicity of V-306 was also tested in New Zealand White rabbits at a dose of 140 μg in PBS. Rabbits were immunized three times SC on days 0, 28, and 56 with V-306 or PBS, and bled on days 0, 14, 38, and 66. The rabbits showed strong immune responses against V-306 with ELISA IgG end-point titers of 5.7 ± 0.2 log_10_ and 5.2 ± 0.2 log_10_ on day 38 and day 66, respectively. Titres of nAbs were tested by 60% PRNT and microneutralization (MN) assays (Fig. 3c). Day-66 rabbit sera showed mean PRNT titers of 5.6 ± 0.9 log_2_ against RSV A2, and 5.1 ± 0.6 log_2_ against RSV A/Long. The MN assay yielded 50% MN titers in the range of 6.9 to 10.4 log_2_ against a panel of recombinant RSV A strains (A2, 2–20 F/G, 91-09, 12–21, 2–20 G-stop) and three recombinant RSV B strains (NH, 9320, TX-1156) (Fig. 3c).

These results demonstrate that V-306 is highly immunogenic in mice and rabbits and highlight the potential of V-306 to elicit strong, long-lasting RSV nAb responses.

### Immunization with V-306 protects lungs from RSV replication

**Fig. 4:**
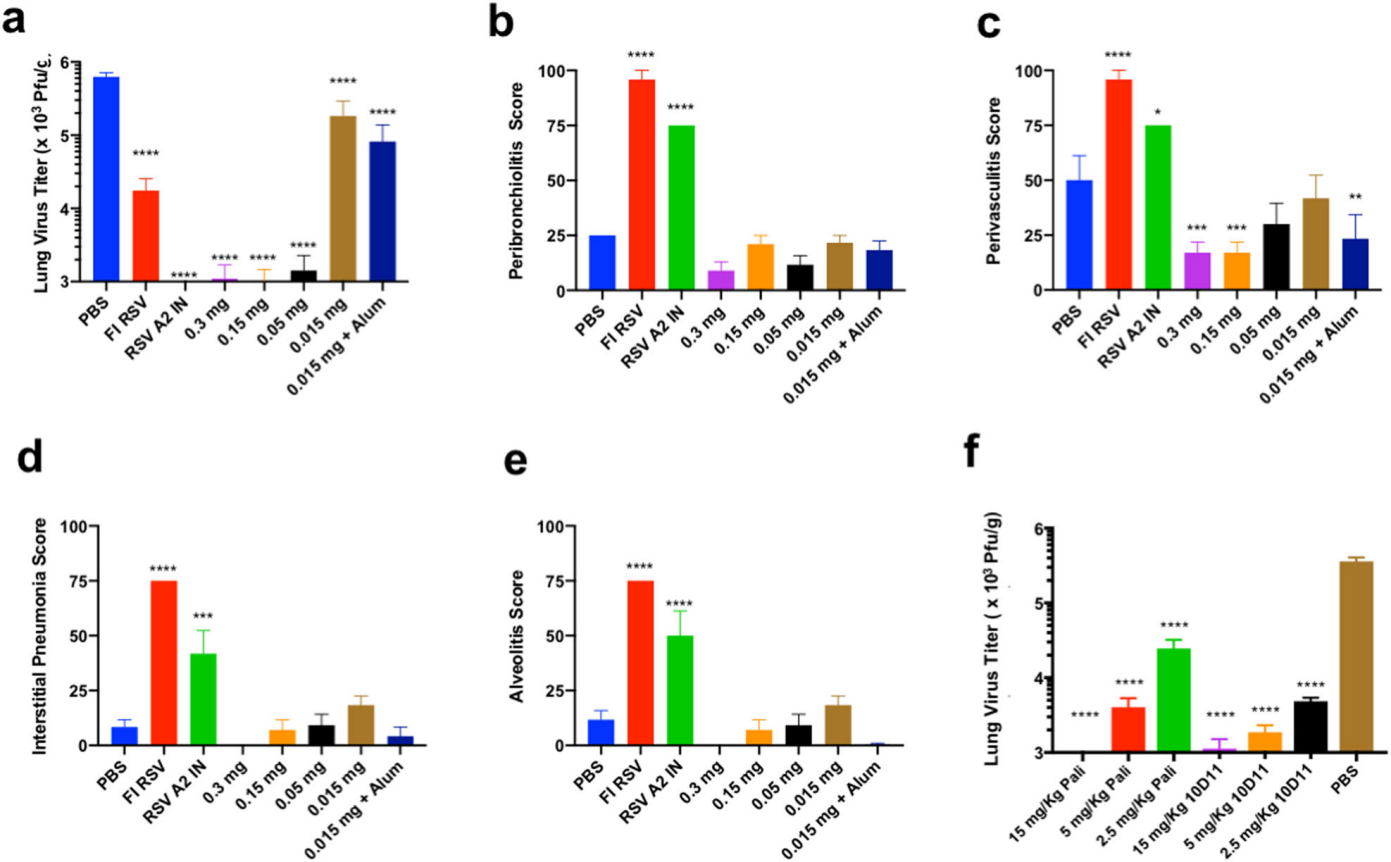
RSV challenge studies. **a** To determine the efficacy of V-306 at different dose levels, BALB/c mice (*n* = 6 per group) were immunized IM twice on days 0 and 21 with the indicated dose levels of V-306 in PBS or Adju-Phos^®^ (Alum), or with PBS (negative control) or FI-RSV (Lot-100 control). An additional group was immunized on day 0 with 10^5^ PFU RSV A2 (positive control). All animals were challenged on day 42 with 10^6^ PFU RSV A2 and sacrificed five days later. Lungs were removed and the virus replication in lungs was analyzed by plaque assay. Lung virus titers are expressed as Pfu/g (****P < 0.0001 vs PBS). **b** Lung pathology was determined by scoring of lung sections for peribronchiolitis, perivasculitis, interstitial pneumonia, and alveolitis. Average peribronchiolitis scores are presented on a 0–100% scale (****P < 0.0001 vs. PBS). **c** Average perivasculitis scores (****P < 0.0001, ***P = 0.0009, **P = 0.0072, *P = 0.0136 vs. PBS), **d** Average interstitial pneumonia scores (****P < 0.0001, ***P < 0.0004 vs. PBS). **e** Average alveolitis scores (****P < 0.0001 vs PBS). **f** Anti-V-306 mAb 10D11 was tested in a passive transfer challenge model in cotton rats (*n* = 5). Different doses of 10D11, palivizumab (positive control), or PBS (negative control) were administered 24 h prior to challenge with 10^4^ PFU of RSV strain A2. Lung titers on day 5 post-challenge were determined by plaque assay (****P ≤ 0.0001 vs. PBS). All *P* values were determined using the Mann–Whitney nonparametric test.

To determine the capacity of V-306 to protect from RSV disease we conducted a challenge study in a validated preclinical RSV-A2 challenge model. Six groups of BALB/c mice (*n* = 6 per animals group) were injected IM with two doses of either 15, 50, 150, or 300 µg V-306 in PBS, 15 µg V-306 + Alum in PBS, PBS alone (negative control) or FI-RSV (Lot-100 control) on days 0 and 21. An additional group of six BALB/c mice was immunized intranasally with 10^5^ PFU RSV A2 (positive control). All groups were challenged intranasally on day 31 with RSV A2 (10^6^ Pfu). The animals were sacrificed 5 days after the challenge, lungs were harvested, weighed, homogenized, and the virus titer was determined for each animal by plaque assay. Mean lung virus titers (expressed as Pfu/g) are shown in Fig. 4a. The animals immunized with 50 µg, 150 µg or 300 µg V-306 or RSV A2 showed significantly reduced RSV replication to low-to-undetectable levels. Animals immunized with 15 µg V-306 or FI-RSV showed an approximately fivefold to tenfold reduction in RSV in the lungs vs. the lungs from PBS-immunized mice. The addition of Alum adjuvant in the 15 µg dose group gave only a small improvement relative to immunization with 15 µg V-306 alone. The results show a high level of protection elicited by V-306 in the lungs against RSV.

### Immunization with V-306 does not elicit lung histopathology typical of VAERD

A major risk of RSV vaccine development is the unwanted induction of VAERD. VAERD was observed after immunization with FI-RSV^26^, as well as recombinant prefusion and postfusion F-proteins^27^. To assess the risk of VAERD induction by V-306 immunization, histopathology was performed on the lungs from the RSV-A2 challenged animals (see Supplementary Fig. 6). Four parameters of pulmonary inflammation were analyzed in each lung section: peribronchiolitis, perivasculitis, interstitial pneumonia, and alveolitis. Each parameter was scored blind using a 0–100% histopathology scale, as described previously^26^. Average pathology scores determined for each parameter are shown in Fig. 4b–e. All animals immunized with V-306 or PBS (negative control for VAERD) both had very low histopathology scores. The lowest pathology scores were observed in V-306 immunized animals, indicating the absence of pathology. Animals immunized with 300 or 150 µg V-306 showed significantly lower perivasculitis scores vs. PBS (Fig. 4c). Co-administration of Alum had no impact on the scores for any of the parameters. In contrast to V-306, a strong lung pathology was observed in all lungs of the FI-RSV-immunized animals and the intranasally RSV-A2-immunized animals.

### V-306 elicits antibodies with palivizumab-like properties

To confirm induction of palivizumab-type antibodies and confirm that the observed reduction of RSV in the lung is antibody-mediated, we generated a monoclonal antibody, called 10D11, against V-306 in BALB/c mice. This mAb is an IgG1(kappa) isotype. The nt and deduced amino acid sequence of the VH and VL regions are shown in Supplementary Fig. 7. Apparent affinities of the epitope mimetic FsIIm to both 10D11 and palivizumab were measured by surface plasmon resonance (BIAcore T200 instrument) with BIA evaluation software 3.1. For each mAb, the sensorgrams could be best analyzed using a 1:1 binding model (see Supplementary Fig. 8). The calculated binding constants are given in Table 1. The apparent affinity of FsIIm to 10D11 was significantly higher (ca. 100-fold) than that for binding to palivizumab. The neutralization activity of 10D11 was compared with that of palivizumab in a validated RSV A Tracy microneutralization (MN) assay and a passive transfer cotton rat challenge model. In the MN assay 10D11 neutralized RSV with MN titer 7.0 log_2_ for RSV A Tracy, approximately fourfold higher than for palivizumab (5.0 log_2_). In the passive transfer model, different doses of 10D11, palivizumab or PBS were injected into groups of five cotton rats. Twenty-four hours later, the animals were challenged with 10^4^ PFU of RSV, sacrificed 5 days after the challenge, and virus titers were determined by plaque assay (Fig. 4f). Both, palivizumab and 10D11, significantly reduced RSV replication in the lungs in a dose-dependent manner, indicating that immunization with V-306 elicits antibodies with palivizumab-like properties that can reduce RSV replication in the lung.

**Table 1:** Binding constants for binding of FsIIm to immobilized 10D11 and commercial palivizumab, measured using surface plasmon resonance (BIAcore T200) and BIAcore evaluation software 3.1.

mAb	k_on_ (1/M s)	k_off_ (1/s)	K_D_ (nM)
10D11	3.26 × 10^6^	0.0335	10
Palivizumab	–	–	1080

The kinetic responses were analyzed for binding to immobilized mAb 10D11, and the equilibrium responses were used for binding to palivizumab (see “Methods” section and Supplementary Fig. 8).

## Discussion

Despite many years of development efforts, a safe and effective RSV vaccine is currently not available. Major complications have included the risk of inducing imbalanced cellular immune responses and/or non-nAbs, which may lead to VAERD. The F-glycoprotein, a major target of nAbs, exists in several conformational states. Many F subunit approaches have focused on postfusion F or on structurally undefined conformational states, which limited the relevant immunogenicity. These vaccine candidates induced high titers of F-binding Abs but typically only low titers of nAbs in RSV naïve preclinical models and only twofold to fourfold increases of nAb titers in human clinical trials, which were not sufficient to confer significant protection from RSV infection^6,7,28^. More recently, DS-Cav-1, a prefusion stabilized RSV-F subunit vaccine candidate was developed, which induced a tenfold increase of nAb titers in a phase I trial in humans^29^. A multitude of epitopes of non- or poorly neutralizing antibodies are present in F, which may limit the boosting of highly potent nAbs in humans and/or induce Abs that block the binding of nAbs to the virus^30^. A significant boost of preexisting nAb responses is particularly important for maternal immunization, aiming to protect infants from respiratory viruses via placental transfer of nAbs^31^.

We address these challenges here by using an epitope-specific structure-guided vaccine design approach, together with a highly immunogenic synthetic nanoparticle delivery system. First, atomic-level structural information^12^, supported by sequencing data from palivizumab-selected MARM viruses^22,23^, was used to design a conformationally constrained peptide FsIIm mimicking the structure of the Motavizumab conformational epitope. Structure determination by NMR spectroscopy of FsIIm showed that it adopts a stable conformation in solution, which closely mimics the 3D structure of the target epitope (Fig. 1). Further optimization gave the constrained peptide V-306p ready for conjugation to synthetic lipopeptides as a potential immunogen (Fig. 1). To verify that V-306p is antigenic, we tested its binding to palivizumab and serum Abs from a panel of 20 randomly selected human sera (Supplementary Fig. 5). In these assays, palivizumab as well as Abs in all tested sera bound specifically and at low concentrations to the V-306p peptide, indicating preservation of the antigenically relevant conformation in the constrained peptide.

The V-306p mimetic was then conjugated to a synthetic lipopeptide that contains a coiled-coil domain and a universal T-helper epitope (Fig. 1 and Supplementary Fig. 2). The resulting lipopeptide (V-306) spontaneously self-assembles into chemically defined micelle-like nanoparticles in PBS with the epitope mimetic displayed in a multivalent format over the surface of the nanoparticle. Such nanoparticles have previously been called Synthetic Virus-Like Particles (SVLPs), because their shape (spherical), size (20–30 nm diameter) and composition (peptide/lipid) resemble such properties of some natural viruses^18,20^. SVLPs have been shown to elicit high titers of epitope-specific Abs in animal models, without the need for an external adjuvant^19,19,21,32^. The V-306 lipopeptide spontaneously formed well-dispersed nanoparticles in PBS with a diameter of ca. 25–30 nm by DLS and EM (Supplementary Fig. 4), which bound to palivizumab in ELISA, indicating that the conformational epitope remained intact on the nanoparticle surface. Immunization of mice and rabbits with V-306 elicited palivizumab-competing Abs (Fig. 2) and very high titers of antigen-specific IgG and RSV nAbs (Fig. 3). Moreover, a single immunization sufficed to elicit long-lasting nAb responses without co-administration of an adjuvant (Fig. 3).

To determine the capacity of V-306 immunization to protect from RSV replication in the mouse lung without causing VAERD, we conducted challenge studies in a preclinical RSV-A2 challenge model. Lungs from V-306 immunized mice, taken 5 days post virus challenge, had very low-to-undetectable virus titers and showed no signs of lung pathology, indicating the absence of VAERD, whereas virus titers in the lungs of PBS- or FI-RSV-immunized animals were high (Fig. 3). Moreover, the lungs from FI-RSV or live RSV immunized mice showed strong signs by histology of enhanced respiratory disease after challenge with RSV A2, whereas the corresponding scores were very low in V-306 immunized animals (Fig. 4).

To confirm that the observed reduction of virus replication in the lungs of V-306-immunized mice is mediated at least in part by palivizumab-like nAbs, we generated a mAb 10D11 from the spleen of a V-306-immunized mouse. 10D11 showed fourfold higher potency than palivizumab by MN assay and reduced lung virus titers in a dose-dependent manner in a passive transfer challenge model.

This is the first successful example of a peptide-based vaccine candidate designed to exclusively target a defined, structurally conserved, protective epitope on RSV. These results encourage us to explore further the clinical development of V-306, including using an epicutaneous route for administration^33^. A phase I clinical study has now been initiated (clinical trials identifier NCT04519073), to provide data on the safety and immunogenicity of V-306 in humans.

## Methods

### Peptide synthesis

Peptides were synthesized by solid-phase peptide synthesis using Fmoc-chemistry and Rink amide resin, using procedures published previously^18,19,32^. For the synthesis of FsIIm, the completed peptide chain was acetylated at the N-terminus prior to cleavage from the resin and removal of side-chain-protecting groups, by treatment with trifluoroacetic acid (TFA), thioanisole, H_2_O, ethanedithiol (87.5:5:5:2,5) for 2.5 h. The peptide was precipitated and washed with *i*Pr_2_O. For oxidation, the reduced peptide was dissolved in 0.33 M ammonium bicarbonate buffer, pH 7.8, and stirred in air overnight. The peptide was purified by reverse-phase (RP)-HPLC on a preparative C18 column and lyophilized to afford a white powder. Analytical RP-HPLC (Vydac 218TP54, 5 µm, 4.6 mm × 250 mm column, 0–60% MeCN in H_2_O (+0.1% TFA) over 40 min): purity: 90.4%; *t*_R_ = 25.07 min. ESI-MS: mass calculated for C_135_H_227_N_43_O_49_S_4_: 3349.52; m/z [M + 3H]^3+^ 1117.51.

For the synthesis of V-306, Bis-Boc-aminooxyacetic acid N-hydroxysuccinimide ester (Boc_2_-Aoa-OSu) was coupled to the N-terminus of the peptide chain. Removal from the resin, deprotection, and oxidation to give V-306p were as described above for FsIIm (Supplementary Fig. 2). The disulfide cross-linked peptide was then purified by RP-HPLC on a preparative C18 column and lyophilized to afford a white powder. Analytical RP-HPLC (Waters BEH C_8_, 1.7 µm, 2.1 × 150 mm column, 10–50% MeCN in H_2_O (+0.05 % TFA) over 45 min, 0.2 mL/min, 30 °C): purity: 92.8%; *t*_R_ = 29.95 min. ESI-MS: Mass calculated for C_135_H_230_N_44_O_49_S_4_: 3379.57 Da; m/z [M + H]^+^: 3380.60 Da (±0.3%).

Linker was prepared by first reacting N-hydroxysuccinimidyl-([N-maleimidopropionamido]-hexa-ethyleneglycol ester (SM-PEG_6_, Thermo Fisher Scientific) with aminoacetaldehyde dimethyl acetal (Aldrich) in H_2_O. SM-PEG_6_ (7.6 mg, 12.6 µmol) was suspended in H_2_O (0.3 mL) and a solution of aminoacetaldehyde dimethyl acetal in H_2_O (17 µl of a 1:10 (v/v)) was added. The mixture was stirred for 90 min at room temperature. The product was purified by RP-HPLC on a C8 column and lyophilized. Analytical RP-HPLC Waters BEH C_8_, 150 × 2.1 mm, 1.7 µm, 0–20% MeCN in H_2_O (+0.05% formic acid) over 20 min, 0.4 mL/min, 40 °C: purity 94.6%, *t*_R_ = 14.29 min. ESI-MS: monoisotopic mass C_26_H_45_N_3_O_12_: 591.30 Da; [M + H]^+^ found: 591.62 Da (±0.1%). Just before conjugation, hydrolysis of the dimethyl acetal was performed with 95% TFA, 5% H_2_O for 5 min. The TFA was removed in vacuo to give the linker. ESI-MS C_24_H_39_N_3_O_11_: 545.26 Da; [M + H]^+^ found: 545.28 Da (±0.05%).

The lipopeptide was synthesized and purified by RP-HPLC, as described elsewhere^18–21^. Analytical RP-HPLC Waters BEH C_8_, 150 × 2.1 mm, 1.7 µm, 64–91% MeOH in H_2_O (+0.05% TFA) over 37.5 min, 0.4 mL/min, 70 °C: purity 97.0%, *t*_R_ = 21.80 min. ESI-MS: monoisotopic mass C_312_H_552_N_74_O_89_S_3:_ 6856.0 Da; m/z [M + H]^+^ found 6860.0 Da (±0.05%).

To prepare V-306, a solution of peptide V-306p (12 mg, 3.6 µmol) in 0.25 ml 0.1 M sodium acetate buffer, pH 3.5 was added to linker (3.8 mg, 7.2 µmol) in 0.25 ml 0.1 M sodium acetate buffer, pH 3.5. The mixture was stirred for 2.5 h and the product oxime (called VMX-3067, Supplementary Fig. 2) was purified by RP-HPLC on a preparative C8 column. Analytical UPLC (Waters BEH C_8_, 1.7 µm, 2.1 × 150 mm, 10 to 40% MeCN in H_2_O (+0.05% formic acid) over 37.5 min, 0.4 mL/min, 26 °C: purity 95%, *t*_R_ = 21.5 min. ESI-MS: mass calculated for C_158_H_263_N_47_O_59_S_4_: 3893.32 Da; m/z [M + H]^+^ found 3893.48 (±0.3%). The oxime (4.0 mg, 1.0 µmol) was dissolved in 0.5 ml H_2_O and added to a solution of lipopeptide (6.2 mg, 0.9 µmol) in 2 ml 50% MeCN. The pH was adjusted to pH = 6.5 with 0.1 N NaOH/0.1 N HCl and the mixture was stirred at room temperature for 2.5 h. The conjugate V-306 was purified by RP-HPLC on a C8 column. The TFA salt was converted first to an acetate salt and then to a hydrochloride salt using AG-X2 anion exchange resin. The conjugate was analyzed by analytical UPLC and MS (Supplementary Fig. 3). UPLC (Waters BEH C8, 1.7 µm, 2.1 × 150 mm) with 20 to 80% MeCN in H_2_O (+0.03% TFA) over 60 min, 26 °C: purity 90%, *t*_R_ = 51.5 min. ESI-MS: monoisotopic mass calc. for C_470_H_815_N_121_O_148_S_7_: 10746.9 Da; m/z [M + 13H]^13+^ found 827.6875 Da (± 0.1%). Conjugate V-306 was suspended in PBS, equilibrated for 30 min, diluted to 1.0 mg/ml, and analyzed by DLS on a DynaPro Nanostar instrument (Wyatt Technology) at 25 °C (Supplementary Fig. 4). The size distribution by regularization analysis was monomodal. The mean hydrodynamic radius (R_h_) was ca. 13 nm, and the polydispersity (Pd) index was 0.038.

### Solution NMR studies

^1^H NMR measurements on FsIIm were performed in H_2_O/D_2_O (9:1) or pure D_2_O, pH 5.0, on a Bruker AV-600 spectrometer at 300 K. Spectral assignments were made using 2D DQF-COSY, TOCSY (mixing time 100 ms) and NOESY (mixing time 250 ms) spectra (see Supplementary Fig. 1). Spectra were typically collected with 1024 × 256 complex data points zero-filled prior to Fourier transformation to 2048 × 1024 and transformed with a cosine-bell weighting function. ^3^*J*_HNα_ coupling constants were determined from 2D NOESY spectra by inverse Fourier transformation of in-phase multiplets. Distance restraints were obtained from 2D NOESY spectra. Additional restraints were introduced for two disulfide bonds between residues Cys4–Cys25 and Cys8–Cys21. The structure calculations were performed by restrained molecular dynamics in CYANA^34^, using the NOE-derived distance restraints and disulfide bond restraints. A final bundle of 20 conformations was selected that incur the lowest CYANA target energy function, shown in Fig. 1b (see also Supplementary Fig. 1).

### Animal studies

Animals studies were performed at Sigmovir Biosystems Inc. (MD, USA). Animals were housed in large polycarbonate cages and were fed a standard diet of rodent chow and water. The colony was monitored for antibodies to adventitious respiratory viruses and other common rodent pathogens and no such antibodies were found. All studies were conducted under applicable laws and guidelines and after approval from the Sigmovir Biosystems, Inc.’s (MD, USA) Institutional Animal Care and Use Committee (IACUC). Production of polyclonal and monoclonal antibodies was performed at Eurogentec SA (Belgium). Animal facilities are accredited by the Belgian authorities. The facilities comply with the highest standards for animal welfare, including accredited by FELASA (Federation of European Laboratory Animal Science Associates), BELAC (Belgium), and the UK Home Office Animals Scientific Procedures Act, and in compliance with ISO 13485 for the in vitro production of mAbs.

### Immunizations

For the analysis of antibody responses, 6–8-week-old female BALB/c mice (five to six per group) or New Zealand white rabbits (two per group) were immunized with antigen dissolved in PBS (100 µL for mice, 400 µL for rabbits). Control animals were immunized with PBS (vehicle), formalin-inactivated RSV (FI-RSV), or live RSV A2 (10^5^ pfu). Blood was collected, and the sera were analyzed by enzyme-linked immunosorbent assay (ELISA) and by RSV nAb assays (60% PRNT and MN test) for nAbs against RSV A2 strains.

The murine mAb 10D11 was made by Eurogentec (Belgium) using V-306 as an immunogen. The spleen cells from immunized mice were fused with non-secretor Sp2/0Ag14 (ATCC^®^ CRL8287™) myeloma cells at a 5:1 ratio, in accordance with Eurogentec (Belgium) methods. Culture supernatants were screened for anti-V-306p antibody by an ELISA and later by an RSV A Tracy virus MN test. Positive hybridomas were cloned twice by limiting dilution and expanded. For large-scale mAb production, cloned hybridoma cell lines were cultured in suspension in roller bottles (Greiner, Belgium) containing 650 mL medium, and mAb was purified by protein G high-performance affinity chromatography (GE Healthcare, Belgium). Purified mAb was dialyzed against PBS, sterile-filtered, and stored at −80 °C.

### ELISA

The ELISA was performed with MaxiSorp 96-well microtitre plates (Nunc, Fischer Scientific), which were coated at 4 °C overnight with 5 μl/ml solutions of peptide V-306p in PBS, pH 7.2 in 50 mM sodium carbonate buffer. The wells were washed with PBS containing 0.05% Tween-20 (PBST) and blocked with PBS containing 5% skimmed milk powder for 1 h at room temperature. After blocking, the wells were washed three times with PBST and incubated with serial fourfold dilutions of mouse sera in PBS containing 0.05% Tween-20 and 0.5% skimmed milk powder (MPBST) for 2 h at room temperature, followed by three washes with PBST. The plates were then incubated with HRP-conjugated goat anti-mouse IgG (Sigma, St. Louis, MO), diluted 1:15,000 in MPBST for 1 h at room temp., washed again three times with PBST and incubated in the dark with 3,3′,5,5′-tetramethylbenzidine (TMB) solution (Sigma) for 15 min. The color reaction was stopped by the addition of 0.16 M H_2_SO_4_, and the absorbance in the wells was read at 450 nm on a plate reader. End-point titers were defined as the highest dilution resulting in an OD value twice that of the buffer. IgG titers were also calculated as reciprocal serum dilutions corresponding to half-maximal binding concentrations (EC_50_). Data were analyzed using GraphPad Prism using nonlinear regression to calculate end-point titers.

Competition ELISA to determine palivizumab-competing antibodies were performed essentially as described above, except that instead of fourfold dilutions of sera, serially twofold diluted (1:20 to 1:2560) sera from immunized or naive control mice, premixed with the constant amount of 50 ng/ml palivizumab in MPBST, were added to the plates. Plates were then processed and read as described above. Palivizumab-competing antibody titers, expressed as IC_50_, were calculated by using GraphPad Prism 6.

### Quantification of palivizumab-like Abs in human sera

These ELISA were performed at the Center for Vaccinology (CEVAC), Ghent University and University Hospital, Belgium, based on the methods described above. Briefly, 96-well polystyrene plates were coated with the SVLP carrier, the V-306p peptide, or the SVLP-antigen V-306. The SVLP carrier was neither recognized by palivizumab nor Abs in the human sera. Then 8 twofold serial dilutions of human serum were added. After washing, anti-human IgG Fc mAb-HRP (1/120,000 dilution) was added. After washing, TMB substrate was added. The color reaction was terminated by the addition of 1 N sulphuric acid. After 30 min the OD is read. The optical density for a given sample was used to extrapolate the Ab titer expressed in µg/mL. The coating conditions were optimized using IgG-depleted human serum spiked with known concentrations of palivizumab such that no further increase in signal is observed (saturation point) and no significant signal is seen in the negative control. The sensitivity of the assay was determined using different concentrations of palivizumab (from 0 to 200 µg/mL) and of anti-human IgG HRP-conjugated Ab (see Supplementary Fig. 5). The detection limit was 0.012207 µg/mL palivizumab (0.61 µg/mL in human serum diluted 1/50). The palivizumab-like Ab concentrations found in 20 different human sera (serum samples 001–020) are shown in Supplementary Fig. 5.

### Plaque reduction neutralization test

Plaque reduction neutralization tests (PRNT) for measurement of RSV-neutralization activity were performed at Sigmovir Biosystems Inc. (MD, USA), as described previously^35^. Briefly, for the RSV-neutralization antibody assay (60% plaque reduction neutralization test (PRNT)), sera were heat-inactivated 30 min. at 56 °C, diluted 1:10 with Eagle’s minimal essential medium (EMEM) and serially diluted further 1:4. Diluted serum samples were incubated with equal volumes of RSV/A2 (25–50 pfu) for 1 h at room temperature and inoculated in duplicates onto confluent HEp-2 monolayers in 24-well plates. After 1 h of incubation at 37 °C in a 5% CO_2_ incubator, the wells were overlayed with 0.75% methylcellulose medium. After 4 days of incubation, the overlays were removed and the cells were fixed and stained with 0.1% crystal violet for an hour and then rinsed and air-dried. The corresponding reciprocal nAb titers are determined at the 60% reduction end point of the virus control using the statistics program “plqrd.manual.entry”. The geometric means ± standard error for all samples in a group at a given time were calculated.

### Neutralizing antibodies in rabbits

One New Zealand white rabbit was immunized with V-306 (150 µg dissolved in PBS) and boosted on days 28 and 56. Serum was collected on day 66. The nAb assays were performed in the laboratory of Dr. Martin Moore at Emory University, USA. A panel of mKate2-tagged RSV chimeric viruses was generated containing the F and G proteins of five A and three B strains, as described earlier^35–37^. For the assays, 96-well plates of HEp-2 cells were prepared. Serum samples were heat-inactivated at 56 °C for 30 min. Serum dilutions of each virus from the clinical panel were prepared in PBS and then added to diluted sera and incubated at 37 °C for 1 h. A sample of the virus/serum mixture was transferred to the HEp-2 plate and incubated for 30 min at 4 °C. A solution of 0.75% methylcellulose dissolved in MEM was added to each well, and the plates were incubated at 37 °C for 48 h before counting FFU per well. The numbers of FFU counted in internal duplicates were averaged. The max. number of FFU counted was taken as 0% and 0 FFU as 100% neutralization. Data analysis used GraphPad Prism. Curve fitting was by nonlinear regression analysis and the EC_50_ was plotted with the upper limit of the 95% confidence interval (Fig. 3c).

### RSV-neutralizing antibodies by microneutralization assay

The RSV A Tracy MN assay was performed by Prof. Pedro Piedra at Baylor College of Medicine, Texas, USA. Heat-inactivated serum samples were analyzed for nAbs against RSV A/Tracy in HEp-2 cells using qualified MN assays as previously described^38–41^. nAb titers were defined as the final dilution at which there was a 50% reduction in viral cytopathic effect. Any serum sample resulting in a titer less than the lower limit of detection (LOD) (2.5 log2) was assigned a value of 2 log2.

### RSV challenge in mice

Challenge experiments were performed at Sigmovir Biosystems Inc. (MD, USA) as described earlier^35^. For determination of protection against challenge, groups of six mice were immunized with 150 µg V-306 in 0.1 ml PBS subcutaneously as described above. Control groups were immunized intramuscularly with FI-RSV or PBS. Twenty-one days following the last immunization, animals were challenged intranasally with 50 µl of RSV A2 at 10^6^ PFU per animal. Five days after the challenge, animals were euthanized, terminally bled for determination of neutralizing antibodies by 60% PRNT as described above and the lungs were harvested and bisected for viral titrations and histopathology analysis.

For viral titrations, lung homogenates were clarified by centrifugation and diluted in EMEM. Confluent HEp-2 monolayers were infected in duplicates with diluted homogenates in 24-well plates. After 1 h of incubation at 37 °C in a 5% CO_2_ incubator, the wells were overlaid with 0.75% methylcellulose medium. After 4 days of incubation, the overlays were removed and the cells are fixed and stained with 0.1% crystal violet for 1 h and then rinsed and air-dried. The plaques were counted and virus titers were expressed as plaque-forming units per gram of tissue. Non-detectable virus was expressed as <2.30 log_10_ PFU/gram.

### Pulmonary histopathology in mice

Pulmonary histopathology studies were performed at Sigmovir Biosystems Inc. (MD, USA) as described previously^35,42^. Briefly, for pulmonary histopathology, lungs were harvested and scored for histopathology as described elsewhere^26^. Lungs were dissected and inflated with 10% neutral buffered formalin to their normal volume, and then immersed in the same fixative solution. Following fixation, the lungs are embedded in paraffin, sectioned, and stained with hematoxylin and eosin (Supplementary Fig. 6). Four parameters of pulmonary inflammation are evaluated: peribronchiolitis (inflammatory cell infiltration around the bronchioles), perivasculitis (inflammatory cell infiltration around the small blood vessels), interstitial pneumonia (inflammatory cell infiltration and thickening of alveolar walls), and alveolitis (cells within the alveolar spaces). Each parameter was scored separately for each histopathologic section with a maximum value of 4 and a minimum of 0^26^. The scores were subsequently converted to a 0–100% histopathology scale.

### Passive transfer comparison of 10D11 and palivizumab

The challenge study comparing 10D11 and palivizumab was performed at Sigmovir Biosystems Inc. (MD, USA). Young adult male cotton rats (8, 6–8 weeks old) were treated IM with the mAb (1.0, 2.5, 5.0, or 15 mg doses). All animals were challenged with 100 µl RSV/A2 at 10^5^ pfu per animal. After sacrificing the animals, nasal and lung tissue were harvested for viral titration using the RSV/A2 60% PRNT assay described above.

### BIAcore experiments

These measurements were performed by Dr. Jens Sobek, at the Functional Genomics Center, University and ETH Zurich. The binding of FsIIm to both 10D11 and palivizumab was measured using a T200 BIAcore instrument (Cytiva USA, formerly GE Healthcare). The mAb was captured on a CM5 sensor chip using a commercial mAb capture kit (Cytiva USA) designed for either a mouse or a human mAb, following the instructions provided by the manufacturer. The capturing Ab was immobilized by standard amine coupling and binds an epitope in all classes of the CH2 domain of IgG Fc. Sensorgrams (Supplementary Fig. 8) were evaluated using the BIA evaluation software 3.1, using a kinetic analysis for 1:1 binding for 10D11, and a thermodynamic analysis for palivizumab, each in HBS buffer (10 mM HEPES, 150 mM NaCl, 3 mM EDTA, 0.005% Tween-20 (polysorbate 20), pH 7.4) at a flow rate of 30 µl/min and 20 °C.

### Reporting summary

Further information on research design is available in the Nature Research Reporting Summary linked to this article.

## Supplementary information

Supplementary Information

Reporting Summary

## Data Availability

Data generated and analyzed during this study are either included in this article and its supplementary information or are available from the corresponding author upon reasonable request.
