# Author Correction: An epitope-specific chemically defined nanoparticle vaccine for respiratory syncytial virus

**DOI:** 10.1038/s41541-021-00375-8

**Published:** 2021-08-27

**Authors:** Armando Zuniga, Oliver Rassek, Melissa Vrohlings, Aniebrys Marrero-Nodarse, Kerstin Moehle, John A. Robinson, Arin Ghasparian

**Affiliations:** 1 Virometix AG, Schlieren, Switzerland; 2 Chemistry Department, University of Zurich, Zurich, Switzerland; 3 Present Address: Shape Biopharmaceuticals Inc, Cambridge, MA USA; 4 Present Address: CDR-Life, Schlieren, Switzerland

**Keywords:** Peptide vaccines, Viral infection

Correction to: *npj Vaccines* 10.1038/s41541-021-00347-y, published online 18 June 2021

In the original version of this Article, the legend to Figure 2c was inadvertently swapped. This has now been corrected in the PDF and HTML versions of the Article.

