# Supplementary material for: An epitope-specific chemically defined nanoparticle vaccine for respiratory syncytial virus
Source: NPJ Vaccines. 2021 Jun 18;6:85. doi: 10.1038/s41541-021-00347-y (PMC8213762; doi:10.1038/s41541-021-00347-y)

An Epitope-Specific Chemically Defined Nanoparticle Vaccine

for Respiratory Syncytial Virus

Supplementary information

Supplementary Figure 1. **NMR studies of FsIIm**. **A**, Chemical shift assignments for FsIIm. **B**, Statistical analysis of structure calculations. **C**, Hα chemical shift deviations from random coil values, showing mostly negative values typical of helical secondary structure. **D**, 600 MHz ^{1}H NMR spectrum of FsIIm in H_2O/D_2O (9:1), pH 5.0.

A

	$^3J_{HN\alpha}$	NH	H-C(α)	H-C(β)	Others
Asn^{1}	*6.8*	8.39	4.70	2.84, 2.84	NH(δ) 7.00, 7.65
Ser^{2}	*4.4*	8.56	4.24	3.94, 3.99	
Glu^{3}	*5.0*	8.63	4.19	2.08, 2.08	CH_2(γ) 2.39, 2.39
Cys^{4}	*5.0*	8.07	4.23	3.09, 3.21	
Leu^{5}	*5.4*	8.07	3.88	1.51, 1.79	CH(γ) 1.69; CH_3(δ) 0.90, 0.90
Ser^{6}	*6.3*	8.12	4.19	3.96, 3.96	
Leu^{7}	*6.1*	7.86	4.17	1.61, 1.90	CH(γ) 1.76; CH_3(δ) 0.88, 0.88
Cys^{8}	*4.7*	7.96	4.36	3.01, 3.06	
Asn^{9}	*4.8*	7.81	4.48	1.85, 2.85	NH(δ) 6.89, 7.63
Asp^{10}	*7.9*	7.71	4.79	2.80, 2.90	
Nle^{11}	*3.7*	7.38	4.41	1.61, 1.80	CH_2(γ) 1.33, 1.33; CH_2(δ) 0.84, 0.84; CH_3(ε) 0.84
Pro^{12}	-	-	4.85	1.95, 2.30	CH_2(γ) 1.96, 2.13; CH_2(δ) 3.58, 3.78
Ile^{13}	*9.8*	8.01	4.77	2.31	$CH_2(\gamma^1)$ 0.96, 1.45; $CH_3(\gamma^2)$ 0.82; $CH_3(\delta^1)$ 0.74
Thr^{14}	*5.5*	8.11	4.34	4.67	$CH_3(\gamma^2)$ 1.35
Asn^{15}	*4.6*	9.12	4.42	2.90, 2.90	NH(δ) 7.08, 7.78
Asp^{16}	*5.1*	8.74	4.44	2.63, 2.72	
Gln^{17}	*5.8*	7.74	4.03	2.06, 2.31	CH_2(γ) 2.37, 2.48; NH(ε) 6.83, 7.50
Lys^{18}	*5.8*	8.72	3.85	1.92, 1.98	CH_2(γ) 1.46, 1.60; CH_2(δ) 1.70, 1.801; CH_2(ε) 3.08, 3.18; NH_3^+(ζ) -
Lys^{19}	*4.5*	7.79	4.09	1.92, 1.92	CH_2(γ) 1.44, 1.57; CH_2(δ) 1.72, 1.72; CH_2(ε) 2.99, 2.99; NH_3^+(ζ) -
Leu^{20}	*6.1*	7.46	4.10	1.63, 1.79	CH(γ) 1.74; CH_3(δ) 0.89, 0.89
Cys^{21}	*4.2*	8.16	4.18	2.88, 3.49	
Ser^{22}	*4.5*	8.50	4.03	3.84, 3.98	
Asn^{23}	*4.8*	8.26	4.51	2.89, 2.93	NH(δ) 6.89, 7.59
Asn^{24}	*6.4*	8.05	4.68	2.94, 2.94	NH(δ) 6.89, 7.63
Cys^{25}	*3.6*	7.92	4.63	3.18, 3.21	
Gln^{26}	*6.6*	7.98	4.32	2.12, 2.23	CH_2(γ) 2.47, 2.47; NH(ε) 6.88, 7.42
Ser^{27}	*6.3*	8.15	4.47	3.95, 3.95	
Val^{28}	*7.6*	7.94	4.13	2.15	CH_3(γ) 0.97, 0.97
Arg^{29}	*6.9*	7.34	4.32	1.80,1.88	CH_2(γ) 1.63, 1.69; CH_2(δ) 3.22, 3.22; NH(ε) 7.26; NH_2(η) -, -
DAla^{30}	*7.3*	8.33	4.29	1.43	

B

	FsIIm
NOE upper-distance limits	297
Intraresidue	105
Sequential	95
Medium- and long-range	97
Residual target function value (Å^2)	0.88 ± 0.07
Mean rmsd values (Å)	
backbone atoms (residues 4-26)	0.40 ± 0.14
heavy atoms (residues 4-26)	0.82 ± 0.414
Residual NOE violation	
Number > 0.2 Å	8
Maximum (Å)	0.39

C

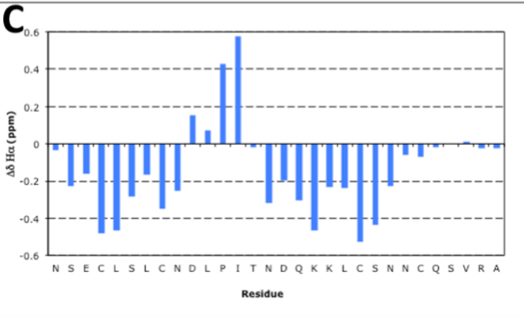

D

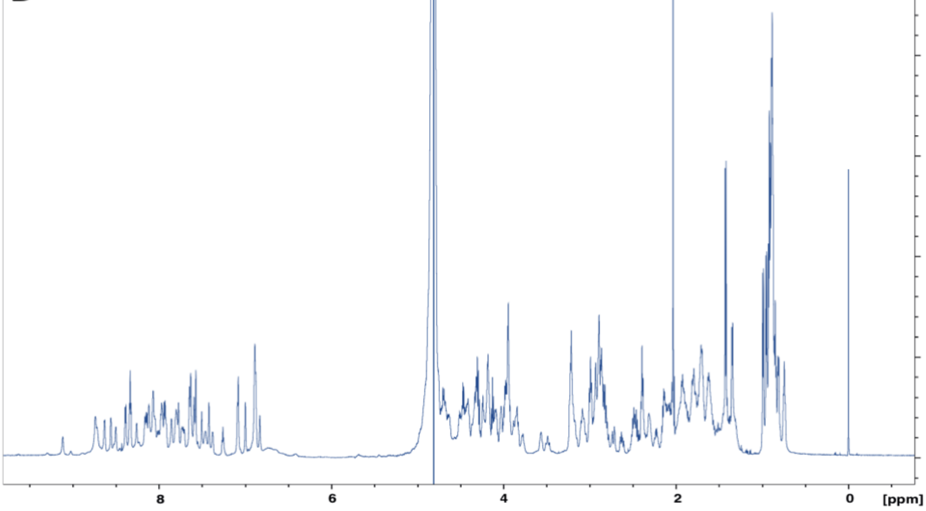

Supplementary Figure 2. **Synthesis of V-306**. The synthetic route to **V-306** (Figure 1) described in the Methods section is shown.

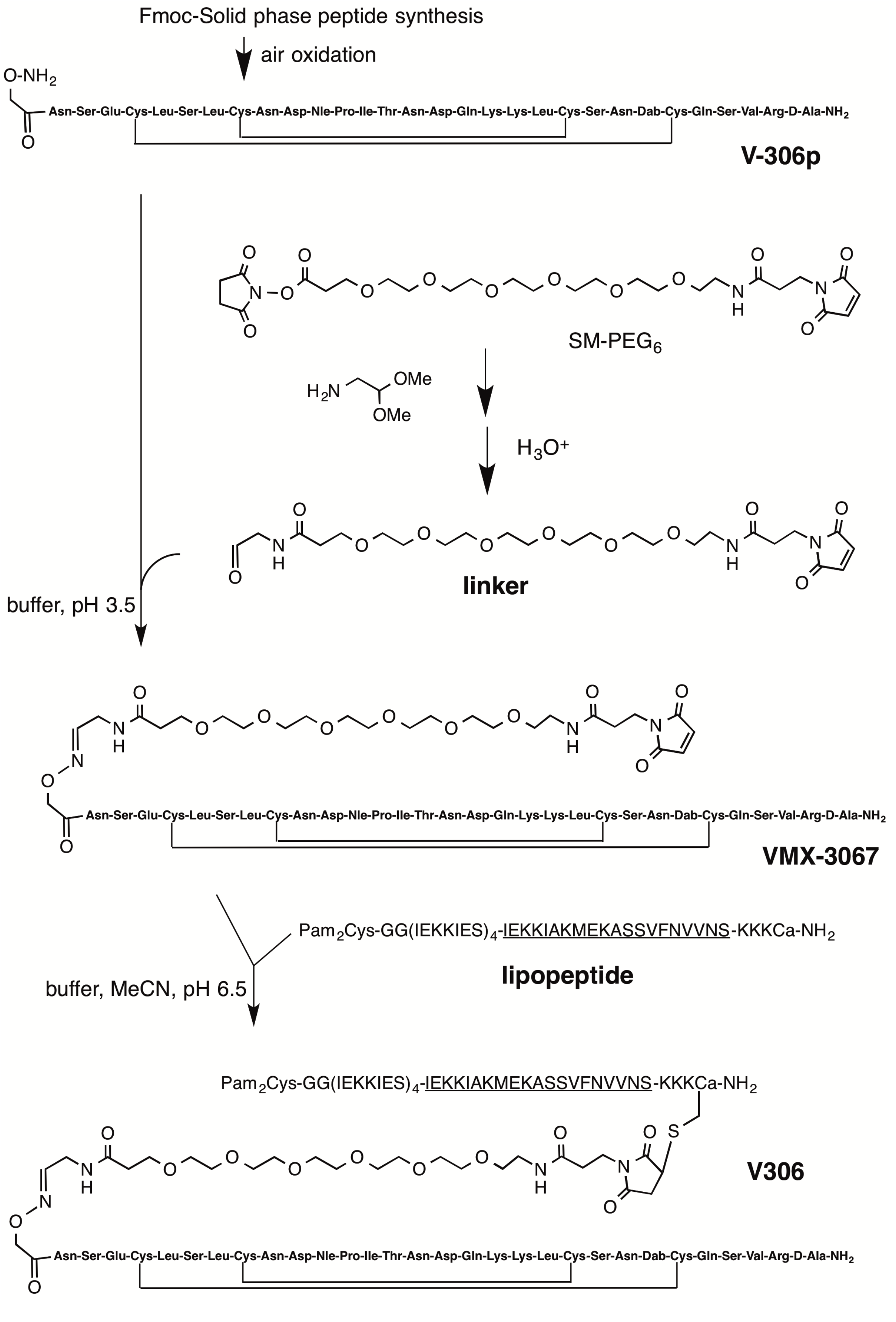

Supplementary Figure 3. **Analytical results for V-306.** *Top*, Analytical UPLC of **V-306** (see Methods section for experimental details). *Bottom*, High resolution electrospray ionization (ESI)-MS of **V-306**.

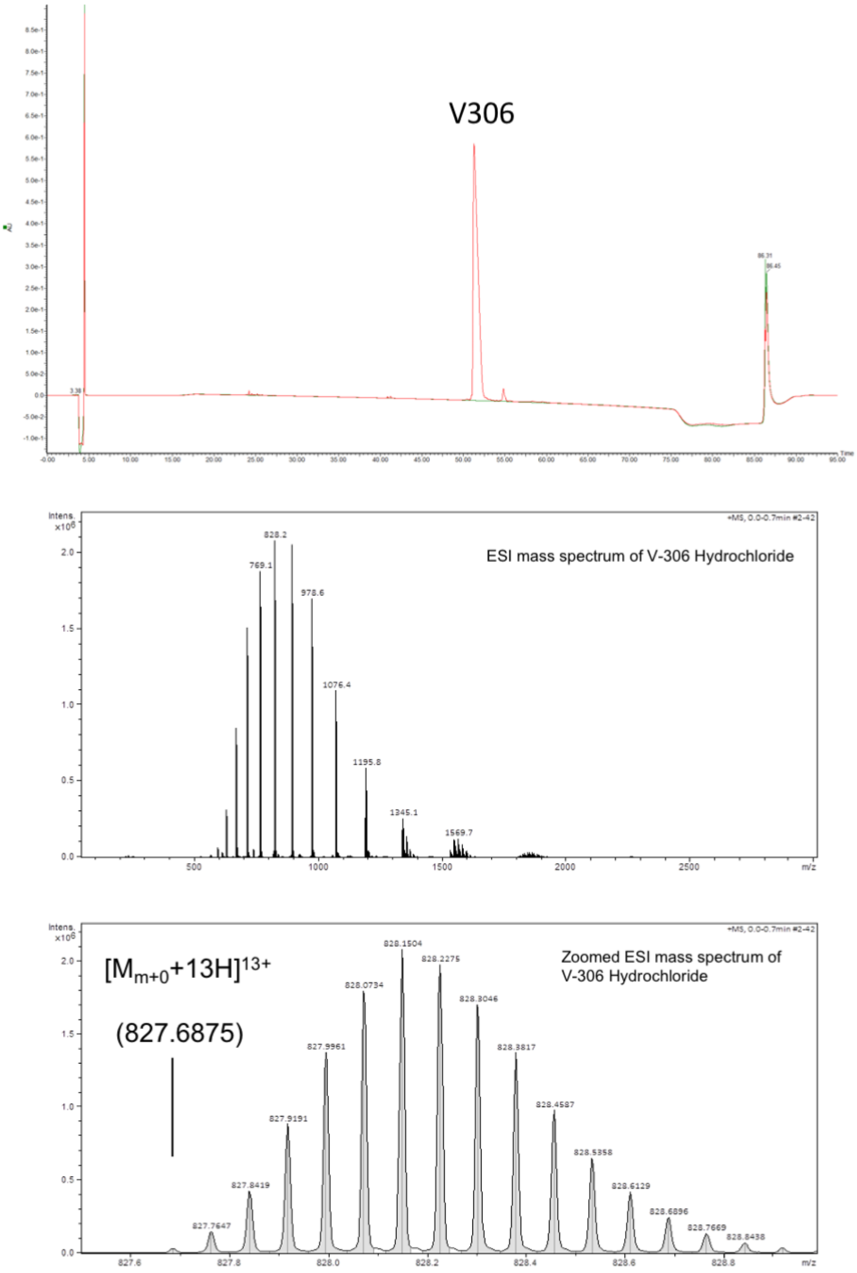

15

Supplementary Figure 4. **EM and DLS data for V-306**. **A,** Negative staining transmission electron micrograph of SVLPs formed by the **V-306** lipopeptide dissolved in tris(hydroxymethyl)amino-methane (Tris) buffer containing 0.9% NaCl, pH 7.4. Method: **V-306** in buffer was applied to glow discharged carbon-coated copper EM grid (400 mesh) and left for 50 s. Excess solution was removed with blotting paper, and then washed with 2 drops of water. The sample was stained with 2% uranyl acetate 3x for 15 min then air dried for 30 min. The sample was then examined by TEM. Scale bar 5x100 nm. **B**, **V-306** lipopeptide was dissolved in PBS (0.5 mg/mL) at pH 7.4, and analyzed in a Wyatt DynaPro DLS instrument at 25°C. Size distributions are shown by regularization as intensity distributions. The average radius, % polydispersity and polydispersity index (PDI) are given below.

A

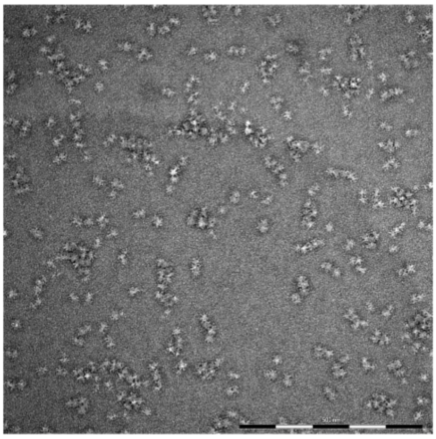

B

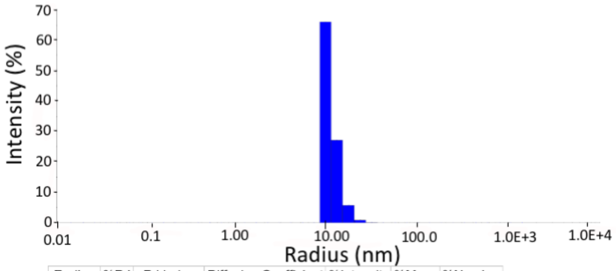

	Radius (nm)	%Pd	Pd Index	Diffusion Coefficient (cm²/s)	%Intensity	%Mass	%Number
Peak 1	10.813	22.8	5.197e-002	2.228e-007	97.6	98.4	100.0
Peak 2	482.870	33.4	1.118e-001	4.990e-009	2.4	1.6	0.0

Supplementary Figure 5. Determination of Abs in human sera recognizing V-306. *Left*, Optimized ELISA calibration for determination of Abs in human sera that bind to peptide (**V-306p**), carrier+peptide (**V-306**) and lipopeptide carrier (shown in Supplementary Figure 2). *Right*, Ab titre in each of 20 different sera. For experimental details see Methods.

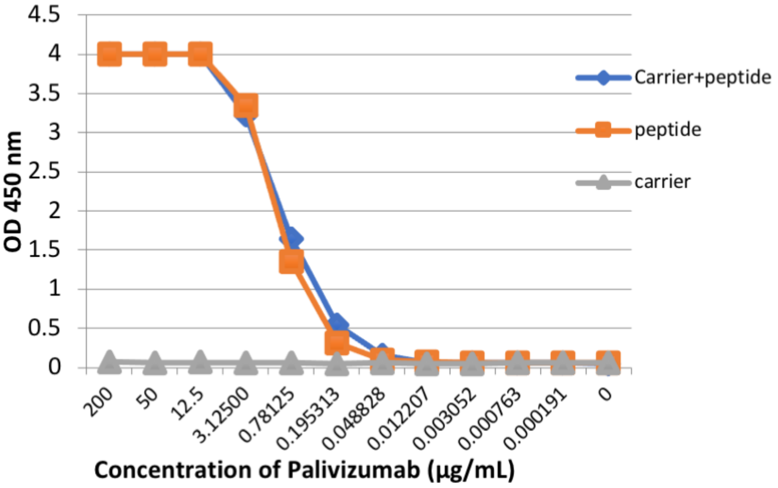

serum Sample	Ab Titer (µg/mL)
001	1,57
002	1,83
003	8,94
004	16,53
005	1,25
006	2,12
007	1,03
008	18,88
009	1,99
010	29,58
011	6,44
012	261,24
013	14,13
014	2,52
015	24,59
016	3,3
017	1,75
018	59,98
019	6,13
020	412,9

Supplementary Figure 6. Representative histopathology pictures. Each picture shows typical histopathology slides following immunizations with different doses of **V-306** (once with adjuvant), with FI-RSV and with PBS (negative control). Scale bar 500 µm.

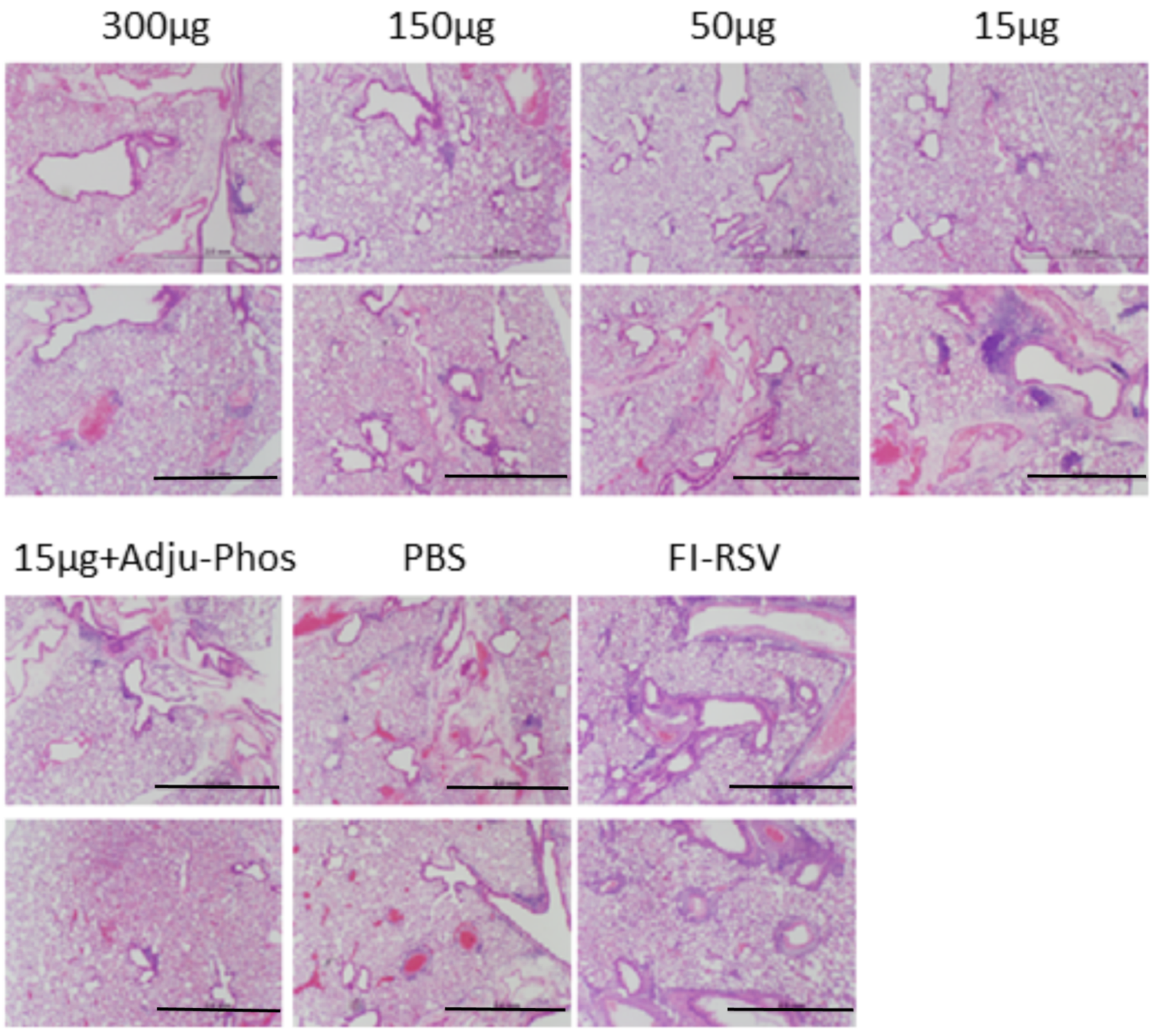

Supplementary Figure 7. Nucleotide and deduced primary sequences of the VH and VL regions of mAb 10D11. The predicted CDRs are underlined.

VH domain

Nt sequence

CAGGTGCAACTGAAGGAGTCAGGACCTGGCCTGGTGGCGCCCTCACAGAGCCTGTCCATTACCTGCA
CTGTCTCTGGGTTCTCATTAACCAGCTATGATATAAGCTGGATTCGCCAGCCACCAGGAAAGGGTCT
GGAGTGGCTTGGAATAATATGGAATGGTGGAGGCACAAATTATAATTCAGCTTTCATGTCCAGACTG
AGCATCAACAAGGACAATTCCAAGAGCCAAGTATTCTTAAAAATGAACAGTCTGCAAACTGATGATA
CAGCCATATATTACTGTGTAAGAGGTGATTATGATTACGCCTGGTTTGATTACTGGGGCCAAGGGAC
TCTGGTCACTGTCTCTGCA

Amino acid sequence

QVQLKESGPGLVAPSQSLSITCTVSGFSLTSYDISWIRQPPGKGLEWLGIIWNGGGTNYNSAFMSRLSINK
DNSKSQVFLKMNSLQTDDTAIYYCVRGDYDYAWFDYWGQGTLVTVSA

VL domain

Nt sequence

AACATTATGATGACACAGTCGCCATCATCTCTGACTGTGTCTGCAGGAGAAAAGGTCACTATGAGCT
GTAAGTCCAGTCAAAGTGTTTTATACGGTTCAAATCAGAAGAACTACTTGGCCTGGTACCAGCAGAA
ACCAGGGCAGTCTCCTAAACTGCTGATCTACTGGGCATCCACTAGGGATTCTGGTGTCCCTGATCGCT
TCACAGGCAGTGGATCTGGGACAGATTTTACTCTTACCATCAGCAGTGTACAAGCTGAAGACCTGGC
AGTTTATTATTGTCATCAATACCTCTTCTCGTGGACGTTCGGTGGAGGCACCAAGCTGGAAATCAAA

Amino acid sequence

NIMMTQSPSSLTVSAGEKVTMSCKSSQSVLYGSNQKNYLAWYQQKPGQSPKLLIYWASTRDSGVPDRF
TGSGSGTDFTLTISSVQAEDLAVYYCHQYLFSWTFGGGTKLEIK

Supplementary Figure 8. Determination of dissociation constants (K_D) using Surface Plasmon Resonance (SPR). A, For 10D11+**Fsllm**. Double referenced SPR sensorgram of interaction of 10D11 with **Fsllm** at concentrations of 3, 6, 12, 24 and 48 nM, with single-cycle kinetic measurement (black) and 1+1 fit (red) using a 1:1 interaction model. **B**, Palivizumab+**Fsllm**. Double referenced SPR sensorgram of interaction of Palivizumab with **Fsllm**. Sensorgrams (black) and fit (red) to a 1:1 binding model, for 8 injections of the peptide in duplicate at concentrations of 7.8, 15.6, 31.25, 62.5, 125, 250, 500 and 1000 nM.

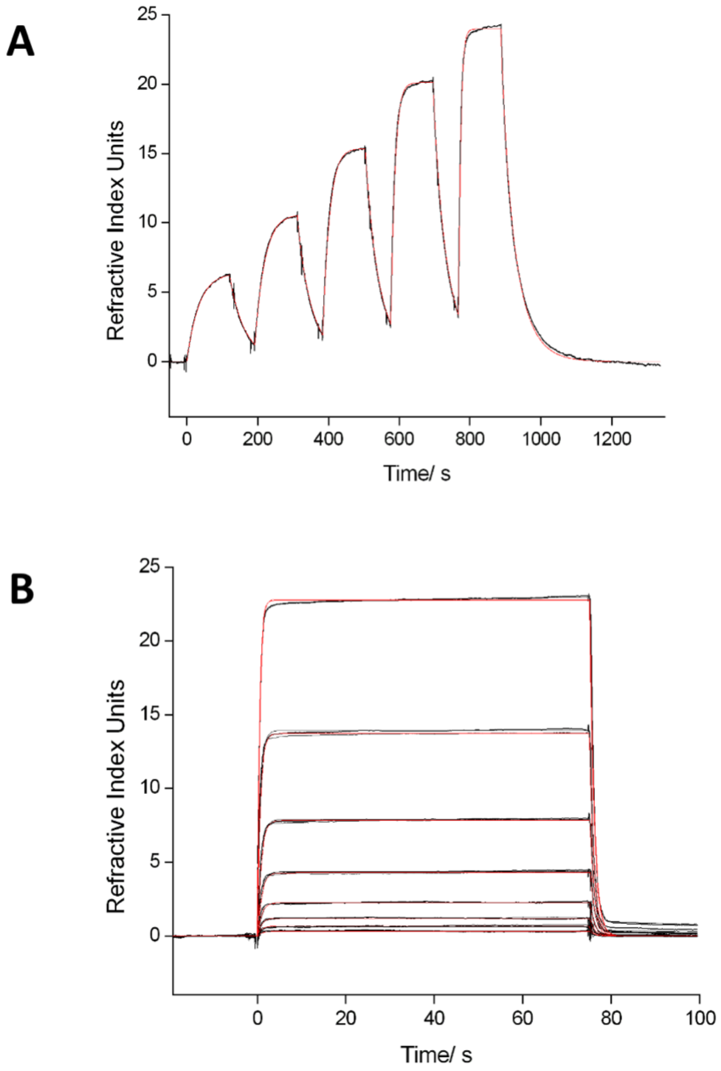

Supplement: Supplementary file 1 — Supplementary Information [file 41541_2021_347_MOESM1_ESM.pdf]
